# Chemical Bonding from the Perspective of *In Situ* Orbital Correlation

**DOI:** 10.1021/acs.jpclett.5c02704

**Published:** 2025-10-09

**Authors:** Xuhui Lin, Yirong Mo

**Affiliations:** † Hunan Key Laboratory of Super Microstructure and Ultrafast Process, School of Physics, 12570 Central South University, Changsha, Hunan 410083, China; ‡ Department of Nanoscience, Joint School of Nanoscience and Nanoengineering, 14616 University of North Carolina at Greensboro, Greensboro, North Carolina 27401, United States

## Abstract

We propose a novel concept of “*in situ*” orbital correlation to gain a deep understanding of the nature of the chemical bond. In stark contrast to popular traditional orbital correlations, where the orbital energies are derived from the free and noninteracting states of isolated species, the “*in situ*” orbital correlations consider the field effects from neighboring species even without any orbital (chemical) interactions. Such field effects may profoundly impact the orbital energies of all of the involved moieties. This is achieved with our block-localized wave function (BLW) method that is the simplest variant of ab initio valence bond (VB) theory and can self-consistently derive a hypothetical diabatic state where the species stay physically together but exclude chemical interactions. Case studies of an exemplary dative bond in H_3_B–NH_3_ and an unconventional ionic bond in lithium–aluminum dimetallocenes demonstrate that the novel “*in situ*” orbital correlation diagram not only provides more insight than the traditional one in general cases but also reshuffles the orbital correlations in cases where the traditional orbital correlation diagram fails.

## Graphical abstract

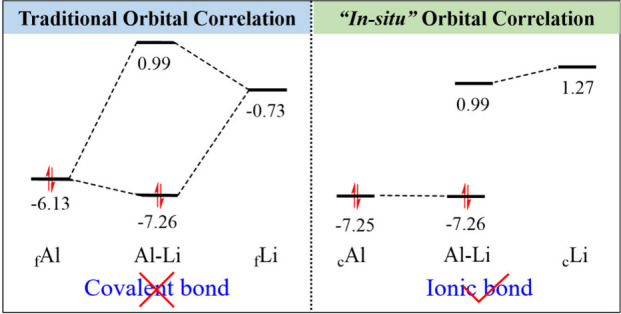

Orbital correlation diagrams are essential for understanding chemical bonding and reactivity, as they map the energy-ordered orbitals of interacting species based on symmetry conservation and dominant orbital compositions.^1–4^ Examples include Walsh diagrams for geometry change^5^ and Woodward–Hoffmann rules for pericyclic reactions.^6^ Currently, the most common type of orbital correlation diagrams is based on frontier molecular orbitals (FMOs), including highest occupied molecular orbitals (HOMOs) and lowest unoccupied molecular orbitals (LUMOs), which play key roles in determining the reactivity and stability in molecules.^7–14^ Most recently, the frontier orbital theory has also been applied to explain reactivity trends in single-atom and alloy catalysis, thereby broadening its relevance to catalysis and surface science.^15–19^

Despite these advances, traditional orbital correlations suffer a fundamental drawback: the orbital energies of interacting species are derived from their free states in isolated geometries. In reality, the presence of interacting species or an external environment, such as solvent, ligands, or catalytic surfaces, can influence orbital energies through electric field, steric, or polarization effects. These subtle but significant perturbations may reshuffle the relative ordering and alignment of frontier orbitals, potentially altering bonding patterns and reactivity in ways not captured by conventional models. To account for these effects, it is desirable to evaluate orbital energies in a hypothetical state in which the interacting species stay “physically” together but without any orbital interactions “chemically” in their complex structure. This concept of a hypothetical state mirrors the diabatic (or resonance) state in the generalized Mulliken–Hush theory of electron transfer.^20,21^ While the diabatic wave functions cannot be directly obtained from standard MO or DFT methods without approximations,^21–23^ it can be well-defined and self-consistently optimized from modern *ab initio* valence bond (VB) theory^24–28^ or approximate approaches such as the transformation from adiabatic states to diabatic states,^29–32^ fragment-based configurations,^33^ constrained DFT,^34^ and block diagonalization.^35^ In particular, the block-localized wave function (BLW) method (details in the Supporting Information),^36–39^ which is the simplest variant of *ab initio* VB theory, can quantitatively derive the wave function for a diabatic state where all orbital mixings or interactions are deactivated with the MO or DFT computational efficiency. Consequently, it can reflect the impact of orbital interactions on molecular geometry, energetics, and spectral properties. For the example of two interacting species A and B, we can construct the wave function Ψ^BLW^ for the intermediate diabatic state as

1 $$\Psi^{BLW}=Â\left(\Phi_{A}\Phi_{B}\right)$$

where all orbitals are block-localized to either A and B, and the orbital energies of A and B in the complex geometry can be subsequently derived. The optimization of orbitals in BLW can be accomplished using successive Jacobi rotation^36^ or Gianinettia et al.’s algorithm.^40,41^ The beauty of the latter lies in the pseudo-Roothaan equation for each block (or species here, *i* = A or B) as

2 $$F_{i}^{\prime }C_{i}=S_{i}^{\prime }C_{i}\varepsilon_{i}$$

where ***F***_*i*_^′^ and ***S***_*i*_^′^ are effective Fock and overlap matrixes with the constraint of ***C***_*i*_^+^ ***S***_*i*_^′^ ***C***_*i*_ = 1. Once we have the optimal block-localized orbitals ***C***_*i*_ derived, we can obtain the orbital energies ***ε***_*i*_ as a diagonal matrix.

Here we propose the concept of “*in situ*” orbital correlations, by introducing an intermediate diabatic state (_c_A and _c_B) between the free species (_f_A and _f_B) and the fully interacting adiabatic state (AB). As shown in Figure 1, this novel concept can illustrate the full evaluation of orbital energy changes from free states to a diabatic state and finally to an adiabatic state. In other words, this approach distinguishes two distinct stages of orbital energy change: (1) from free to diabatic state, capturing nonbonding physical interactions (e.g., electrostatics and Pauli repulsion, or alternatively steric effect, and polarization), and (2) from diabatic to adiabatic state, representing genuine chemical orbital interactions (i.e., covalent bonding).

**Figure 1:**
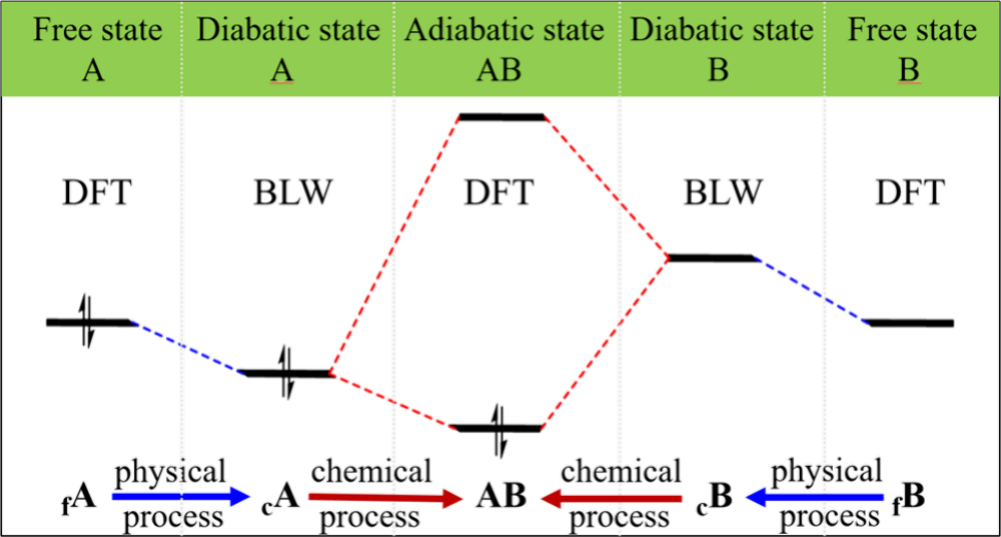
Schematic “*in situ*” orbital correlation diagram. _f_X and _c_X (X = A or B) indicate the free X in an isolated state and a constrained state within the complex diabatic (BLW) state, respectively.

In the following, we studied two exemplary cases, ammonia borane and lithium–aluminum dimetallocene, in an attempt to apply the “*in situ*” orbital correlation concept to gain a deeper understanding of their chemical bonding nature. All computations were performed with the in-house version of the GAMESS software^42^ where our BLW method was ported to. The M06-2X functional^43^ augmented with Grimme’s D3­(BJ) dispersion correction^44^ and def2-TZVPP basis function^45^ were adopted throughout. It should be noted that functional choice has no appreciable effect on the results and analysis (see Table S1 and Figures S1–S6 in the Supporting Information).

We first explored the bonding in H_3_NBH_3_, whose dative B ← N bond can be illustrated simply on the traditional orbital correlation diagram, where the HOMO of NH_3_ and the LUMO of BH_3_ correctly highlights the electron transfer from the Lewis base NH_3_ to the Lewis acid BH_3_. To better understand the orbital interaction, we computed the orbital energies of both NH_3_ and BH_3_ at their complex geometry, with their orbital interactions disabled. It is known that the orbital interaction or electron transfer stabilizes the complex significantly.^46^

When NH_3_ and BH_3_ are put together physically, the HOMO of NH_3_ lowers its energy but the HOMO–1 of BH_3_ which is symmetrically compatible with the HOMO of NH_3_ raises the energy. These shifts dramatically reduce the energy gap between both occupied MOs from 6.92 eV in their free states to only 0.09 eV in the diabatic state. The subsequent mixing between the two orbitals leads to one at a high energy level and one at a low energy level. The one in high energy level further interacts with the LUMO of BH_3_ and gets stabilized. Obviously, the “in situ” orbital correlation diagram framed in red dashed lines in Figure 2 provides more information on orbital interactions than the traditional orbital correlation diagram and thus enriches our understanding of the bonding details.

**Figure 2:**
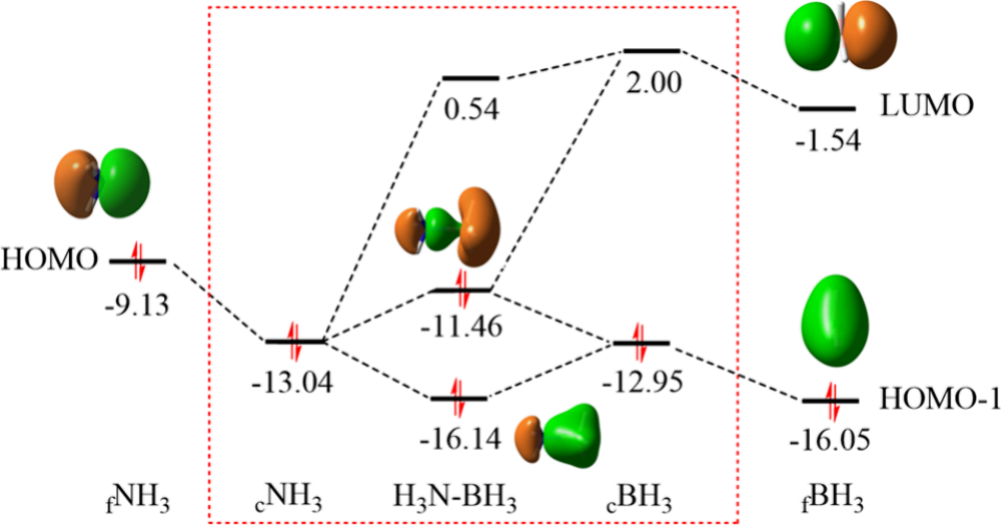
“*In situ*” orbital correlation diagram (in eV) for the dative bond in H_3_N–BH_3_ where orbitals are plotted with isovalues = 0.05 a.u..

While the “*in situ*” orbital correlation diagrams provide a more detailed and enhanced understanding of the dative bond in H_3_N–BH_3_, traditional orbital correlation diagrams remain qualitatively reliable for many similar systems. However, they will simply fail in other cases. A notable example is the recently synthesized lithium–aluminum heterobimetallic dimetallocene, i.e., ^5^CpAl–Li^5^Cp, reported by Schäfer et al. (Figure 3a), where “^5^Cp” refers to an isopropyl-substituted cyclopentadienyl ligand.^47^ In this complex, the Al–Li bond exhibits high ionic character, further stabilized by attractive dispersion interactions between the bulky isopropyl groups. Remarkably, the authors also claimed a considerable covalent component in the Al–Li bond. This covalency is attributed to electron donation from the aluminum lone pair into the vacant 2*s* orbital of lithium, which is a classic donor–acceptor interaction, like in the above H_3_NBH_3_ complex. The covalent bonding character seems well supported by both the orbital correlation diagram (Figure 3b) and energy decomposition analysis-natural orbitals for chemical valence (EDA-NOCV), which reveals an overall orbital interaction energy of −10.42 kcal/mol. Similar bonding patterns have been observed in other dimetallocene derivatives such as CpAl–LiCp and *CpAl–Li*Cp (Cp and *Cp refer to cyclopentadienyl and pentamethylcyclopentadienyl, respectively.), though these analogues lack the pronounced dispersion stabilization due to bulky substituents as in ^5^Cp. Notably, the dimetallocene CpAl–LiCp has already been theoretically predicted and studied, where the donor–acceptor charge transfer was found to play a dominant role in stabilizing the Al–Li bond.^48–50^

**Figure 3:**
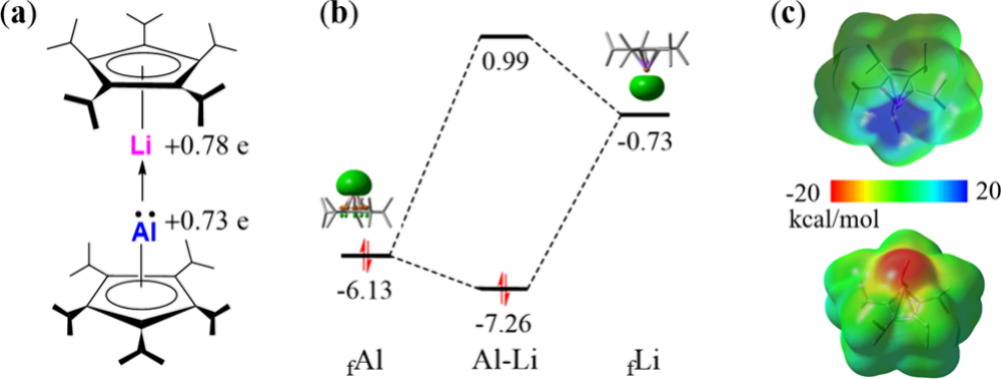
(a) Structure of ^5^CpAl–Li^5^Cp and NPA charges for Al and Li atoms. (b) Molecular orbital correlation diagram (in eV) for the Al–Li σ-bond. (c) ESP maps for ^5^CpAl and ^5^CpLi.

However, the ionic nature of the Al–Li bond seems controversial, especially from the perspective of conventional MO or DFT-based analysis. On one hand, both Al and Li centers carry partial positive charges according to the natural population analysis (NPA, see Figure 3a)^51^ or similar schemes such as the chelpg charges (0.035 and 0.238 for Al and Li, respectively) derived from the electrostatic potential, suggesting that their interaction should be electrostatically repulsive rather than attractive. On the other hand, the traditional orbital correlation diagram (Figure 3b) suggests a dative bonding scenario similar to that in H_3_NBH_3_, with significant charge transfer between ^5^CpAl and ^5^CpLi. Specifically, the HOMO of ^5^CpAl corresponds to a lone pair on the Al-centered *s* orbital, while the LUMO of ^5^CpLi involves a vacant *s* orbital on the Li center. These frontier orbitals are symmetry-compatible and separated by a moderate energy gap of 5.4 eV (7.59 eV in H_3_NBH_3_), implying that orbital-driven donor–acceptor interactions are energetically plausible.

For the first question above, the electrostatic attraction between ^5^CpAl and ^5^CpLi can be best rationalized through the electrostatic potential (ESP) maps as shown in Figure 3c. These maps reveal that the Al atom in ^5^CpAl features a σ-plunge with a concentrated negative electrostatic potential. In contrast, the Li center in ^5^CpLi displays a σ-hole characterized by a region of positive electrostatic potential along the axis of the Al–Li bond. This suggests that while both centers carry partial positive charges overall, the local anisotropy in electron density allows for directional electrostatic attraction that facilitates bond formation.

Yet, despite the orbital alignment and observable orbital interaction energies in EDA, the Al–Li bond does not appear to exhibit a dominant covalent character. In typical EDA schemes,^52–55^ the orbital interaction term contains contributions from both polarization (orbital relaxation within fragment) and charge transfer (orbital mixing between fragments). These two different interactions can be easily decoupled using our BLW-based energy decomposition (BLW-ED, details in the Supporting Information).^56–58^ The BLW-ED analysis in Table 1 shows that the sum of polarization and charge transfer closely matches the total orbital interaction reported in the literature. Nevertheless, only about 10% of total interacting energy originates from the charge transfer from the HOMO of ^5^CpAl to the LUMO of ^5^CpLi, indicating that a direct covalent interaction seems insignificant. We further performed the BLW method to reoptimize the geometries with the Al lone pair strictly localized on itself (thus excluding any dative covalency). For each dimetallocene, the BLW geometries exhibit only slight stretching of the Al–Li bond distances by 0.1 Å. In contrast, the bond distance in dative H_3_NBH_3_ is significantly elongated from 1.649 to 2.358 Å, indicating the dominance of the charge transfer and nonbinding interaction without charge transfer. Therefore, the Al–Li bond is essentially ionic in nature and can be effectively interpreted in purely Coulombic terms, including electrostatics and polarization. The ionic nature of the Al–Li bonds can also be supported by quantum chemical topology method QTAIM^59^ with Multiwfn,^60^ where the topological parameters (see Table S1) suggests that they are closed-shell interactions.

**Table 1:** BLW-ED Components (kcal/mol) and Optimized Bonding Distances (Å) for Lithium–Aluminum Dimetallocenes (*R*_d_ = *R*_Al–Li_) and H_3_N–BH_3_ (*R*_d_ = *R*_N–B_) at the M06-2X-D3/Def2-TZVPP level

Complex	Δ*E*_int_	Δ*E*_disp_	Δ*E*_f_	Δ*E*_pol_	Δ*E*_ct_	*R*_*d*_^DFT^	*R*_*d*_^BLW^
^5^CpAl–Li^5^Cp	–22.03	–3.36	–9.89	–6.36	–2.42	2.630	2.767
*CpAl–Li*Cp	–11.83	–0.72	–4.45	–5.13	–1.40	2.732	2.844
CpAl–LiCp	–9.84	–0.20	–4.18	–4.30	–1.40	2.755	2.851
H_3_N–BH_3_	–46.57	–0.00	23.88	–30.50	–38.35	1.650	2.358

Given the limitations of traditional orbital correlation diagrams in capturing the ionic character of metal–metal bond, we constructed the “*in situ*” orbital correlation diagram for ^5^CpAl–Li^5^Cp in Figure 4, while comparable diagrams for CpAl–LiCp and Cp*Al–Li*Cp are provided in Figures S1 and S2, respectively. It can be seen from Figure 4 that the HOMO of ^5^CpAl is stabilized by the presence of ^5^CpLi from −6.13 eV to −7.25 eV, while the LUMO of ^5^CpLi is destabilized from −0.73 to 1.27 eV. The resulting HOMO–LUMO gap between the donor and acceptor fragments increases substantially from 5.4 to 8.52 eV, indicating a drastically reduced orbital interaction. Although the energy gap between the degenerate LUMOs of ^5^CpAl and the HOMOs of CpLi decreases during the physical interaction, they are not symmetry-compatible, and thus, any orbital interaction is disfavored. Moreover, a comparison between the diabatic (e.g., −7.25 eV for HOMO of ^5^CpAl) and adiabatic (e.g., −7.26 eV for HOMO of ^5^CpAl) molecular orbital energies shows that the occupied MOs remain largely unchanged upon complex formation. These observations agree with the BLW-ED analyses, where the orbital interaction component of the total binding energy is dominated by polarization, with insignificant contribution from the charge transfer interaction. Therefore, the “*in situ*” orbital correlation diagram provides an insightful understanding for the BLW-ED data, showing that the Al–Li bond is perfectly ionic in nature, stabilized through electrostatic and polarization effects.

**Figure 4:**
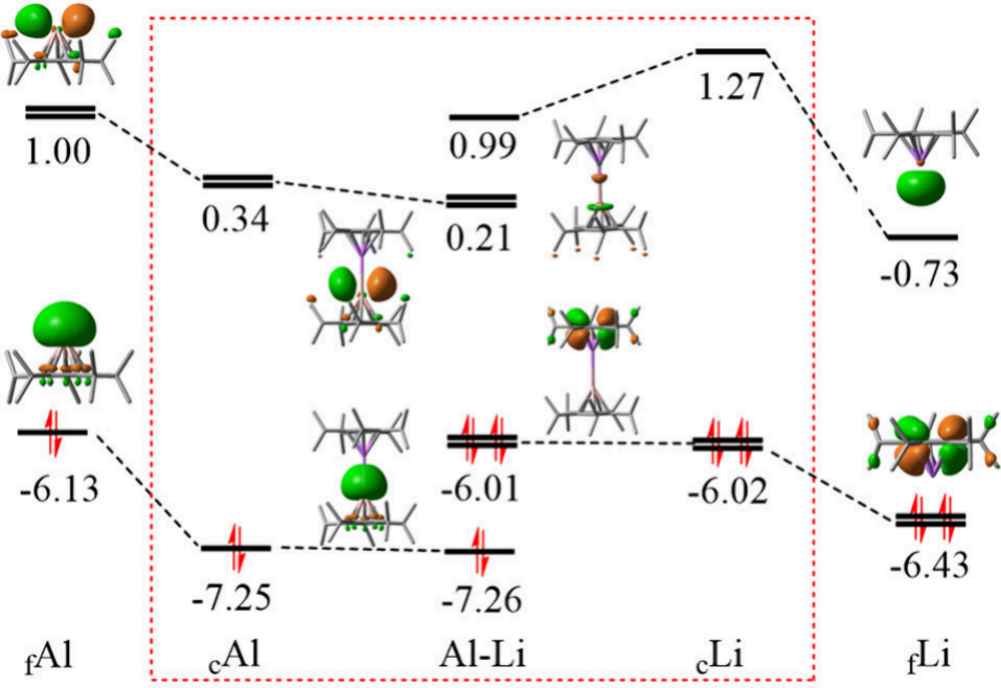
“*In situ*” orbital correlation diagram (in eV) showing the ionic bond between ^5^CpAl and ^5^CpLi with isovalues = 0.05 a.u..

In summary, the proposed “*in situ*” orbital correlation concept is based on the orbital energies of interacting species from a hypothetical diabatic state, which can be derived with the BLW method. Unlike the traditional orbital correlation diagram, the “*in situ*” one differentiates the external field effect imposed by neighboring species from their chemical bonding interactions, thereby offering a more realistic depiction of orbital interactions in chemical bonding scenarios. When applied to a series of Al–Li heterobimetallic dimetallocenes, the “*in situ*” orbital correlation diagrams reveal that the Al–Li bonds are ionic and exhibit insignificant charge transfer between the Al-centered HOMO and the Li-centered LUMO, in contrast to the traditional orbital correlation diagrams, which suggest a potential donor–acceptor interaction. We anticipate that the “*in situ*” orbital correlation will serve as a powerful conceptual and computational tool for the investigation of chemical bonding and electronic structures.

## Supplementary Material



## Notes

Methodology for BLW and BLW-ED, supplemental tables and figures, and *xyz* coordinates of all studied structures (PDF)
