# Supplementary material for: Chemical Bonding from the Perspective of In Situ Orbital Correlation
Source: J Phys Chem Lett. 2025 Oct 9;16(41):10811–5. doi: 10.1021/acs.jpclett.5c02704 (PMC12536439; doi:10.1021/acs.jpclett.5c02704)
Supplement: Supplementary file 1 [file jz5c02704_si_001.pdf]

Supporting Information

Chemical Bonding from the Perspective of *In-Situ* Orbital Correlation

Xuhui Lin*[1] and Yirong Mo*[2]

[1] Hunan Key Laboratory of Super Microstructure and Ultrafast Process, School of Physics, Central South University, Changsha, Hunan 410083, China;

[2] Department of Nanoscience, Joint School of Nanoscience and Nanoengineering, University of North Carolina at Greensboro, Greensboro, NC 27401, United States.

E-mails: xuhui.lin@csu.edu.cn (XL); y_mo3@uncg.edu (YM)

Contents

1. Methodology..S2
2. Table S1-S2..„„„„„„„....S5
3. Figure S1-S6 ....„„„„„.....S6
4. xyz coordinates for Optimal Geometries S10

1. Methodology

1.1 Block-localized wavefunction (BLW) method.

Molecular orbital (MO) theory assumes that all electrons are in MOs which are delocalized to the whole system, whereas valance bond (VB) theory starts from localized atomic orbitals to build Lewis (resonance) structures and describes a conjugated system with several resonance structures.[1-3] Each resonance structure can be defined with a Heitler-London-Slater-Pauling (HLSP) function which is essentially a combination of a number of Slater determinants. To simplify the computational costs and combine the advantages of both MO and VB theories, we proposed the BLW method where a BLW corresponds to a unique electron-localized diabatic state (usually the most stable resonance state).[4,5] The fundamental assumption is that all electrons and primitive basis functions (χ) can be divided into k subgroups (blocks), and each MO is block-localized and expanded in only one block. Assuming that there are m_i basis and n_i electrons for block i, we can express block-localized MOs for this block as

$$\varphi_j^i = \sum_{\mu=1}^{m_i} C_{j\mu}^i \chi_\mu^i \quad (1)$$

Subsequently, the BLW for a closed-shell is defined using a Slater determinant as

$$\Psi^{\mathrm{BLW}} = \det\left|\left(\phi_1^1\right)^2\left(\phi_2^1\right)^2\cdots\left(\phi_{n_1/2}^1\right)^2\cdots\left(\phi_1^i\right)^2\cdots\left(\phi_{n_i/2}^i\right)^2\cdots\left(\phi_{/n_k/2}^k\right)^2\right| = \hat{A}\left[\Phi_1\Phi_2\cdots\Phi_k\right] \quad (2)$$

Orbitals in the same subspace are subject to the orthogonality constraint, but orbitals belonging to different subspaces are nonorthogonal. The BLW method is available at the DFT level with the geometry optimization and frequency computation capabilities.

The BLW method has been extended to the density functional theoretical (DFT) level with the Grimme's dispersion correction incorporated. Furthermore, both geometrical optimization and spectral property calculations of the diabatic state (namely BLW state) are available. The role of charge transfer interaction (or resonance), a fundamental concept in chemistry, can be quantitatively examined by comparing the electron-delocalized (with a regular DFT computation) and electron-localized (with a BLW computation at the same theoretical level) states in terms of energy, electron density distribution, and vibrational modes.

2.1 Energy decomposition (BLW-ED) scheme based on the BLW method

Energy decomposition analysis (EDA) can reveal the contributions of chemically and physically meaningful factors to chemical interactions, offering clear explanation from an energetic perspective. Numerous EDA schemes, such as the Kitaura and Morokuma (KM) method,[6] symmetry-adapted perturbation theory (SAPT),[7-10] EDA-NOCV,[11,12] and GKS-EDA,[13,14] have been developed and extensively applied. Energy decomposition based on the BLW method (BLW-ED in short) has also been developed, offering a chemically intuitive tool for exploring chemical interactions. The two-block model, specifically for a Lewis acid A and a Lewis base B, provides the simplest description of non-covalent interactions and is adopted here to introduce the BLW-ED approach.

In the BLW-ED approach, the interaction energy is divided into the frozen, polarization, charge transfer energy and dispersion components (eq. 3). The frozen energy represents the energy change associated with bringing deformed and infinitely separated monomers to their positions in the complex, without altering their electron densities. The expression for the frozen energy is eq. 4, where Ψ^{BLW0} refers to the initial electron localized state, constructed with optimal orbitals of isolated monomers at their geometries in complex (eq. 5). The complex can be stabilized through adjusting the electron densities within each monomer, driven by the influence of the electric field and Pauli exchange repulsion from the other monomer. This energy-lowering effect corresponds to the self-consistent optimization of the electron-localized wavefunction, and is referred to as polarization, whose stability is measured with the energy difference between the optimized electron-localized state (Ψ^{BLW}) and the initial diabatic state (eq. 6). Subsequently, additional stability can be achieved by allowing all orbital expansion spaces to extend from a specified subgroup to the entire set of basis functions of the complex. This energy change is defined as the charge transfer energy (eq. 7), in which the basis set superposition error (BSSE) is included. Notably, the electron delocalized state is denoted with Ψ^{MO}, representing the regular Hartree-Fock (HF) or DFT wavefunction.

$$\Delta E_{\mathrm{int}} = \Delta E_{\mathrm{F}} + \Delta E_{\mathrm{pol}} + \Delta E_{\mathrm{CT}} + \Delta E_{\mathrm{disp}} \tag{3}$$

$$\Delta E_{\mathrm{F}} = E(\Psi^{\mathrm{BLW0}}) - E(\Psi_{\mathrm{A}}) - E(\Psi_{\mathrm{B}}) \tag{4}$$

$$\Psi^{\mathrm{BLW0}} = \hat{A}(\Psi_{\mathrm{A}}\Psi_{\mathrm{B}}) \tag{5}$$

$$\Delta E_{\mathrm{pol}} = E(\Psi^{\mathrm{BLW}}) - E(\Psi^{\mathrm{BLW0}}) \tag{6}$$

$$\Delta E_{\mathrm{CT}} = E(\Psi^{\mathrm{MO}}) - E(\Psi^{\mathrm{BLW}}) + \mathrm{BSSE} \tag{7}$$

$$\Delta E_{\mathrm{disp}} = \Delta E_{\mathrm{disp}}^{\mathrm{Dimer}} - \Delta E_{\mathrm{disp}}^{\mathrm{A}} - \Delta E_{\mathrm{disp}}^{\mathrm{B}} \tag{8}$$

Although DFT already encompasses most electron correlations, other advanced methods including additional electron correlations are essential for studying non-covalent interactions. Density functionals offer alternative approaches for incorporating electron correlation with notable computational efficiency. In this context, Grimme's D3 dispersion correction plays an indispensable role. Accordingly, the difference in Grimme's D3-dispersion correction between the complex and the sum of deformed monomers is defined as the dispersion correction component (ΔE_{disp} in eq 8), which is a density independent energy component. Furthermore, the electrostatic interaction, Pauli exchange repulsion and partial electron correlation are included in the frozen component at DFT level, and can be obtained using the XEDA method[15] developed by Su et al. due to the low computational cost.

Table S1. BLW-ED components (kcal/mol) at the **B3LYP-D3/Def2-TZVPP** level.

Complex	ΔE_{int}	ΔE_{disp}	ΔE_{f}	ΔE_{pol}	ΔE_{ct}
^{5}CpAl–Li^{5}Cp	-22.16	-13.81	-0.15	-5.51	-2.69
*CpAl–Li*Cp	-13.66	-3.40	-4.43	-4.40	-1.42
CpAl–LiCp	-11.00	-2.19	-3.01	-4.10	-1.67
$H_3N–BH_3$	-43.68	-1.90	28.19	-36.20	-33.77

Table S2. Characterization of the BCPs at the Intermolecular Bonds with QTAIM Analysis at the M06-2X-D3/Def2-TZVPP

Complex	ρ	$\nabla\rho^2$	G	G/ρ	H
^{5}CpAl–Li^{5}Cp	0.0154	0.0462	0.0113	0.7353	0.0002
*CpAl–Li*Cp	0.0125	0.0391	0.0092	0.7367	0.0005
CpAl–LiCp	0.0118	0.0363	0.0085	0.7203	0.0006
$H_3N–BH_3$	0.1045	0.4137	0.1724	1.650	-0.0689

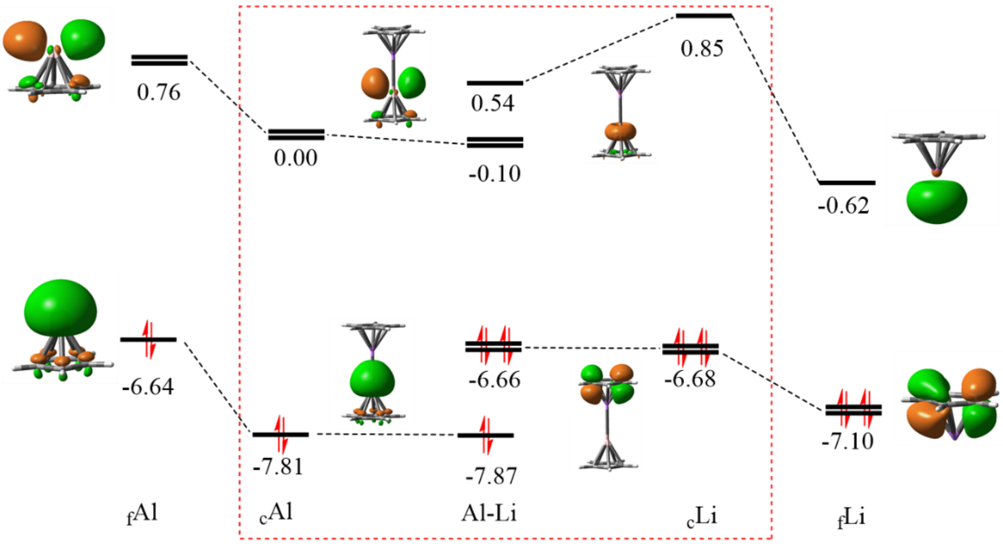

Figure S1. "*In-situ*" orbital correlation diagram with **M06-2X** functional showing the ionic bond between CpAl and CpLi with isovalues = 0.05 a.u..

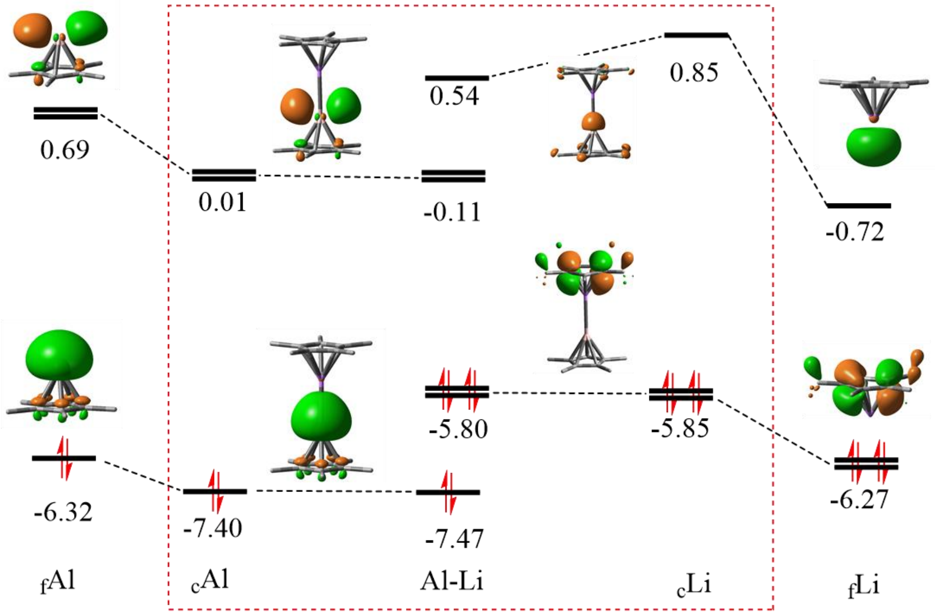

Figure S2. "*In-situ*" orbital correlation diagram with **M06-2X** functional showing the ionic bond between *CpAl and *CpLi with isovalues = 0.05 a.u..

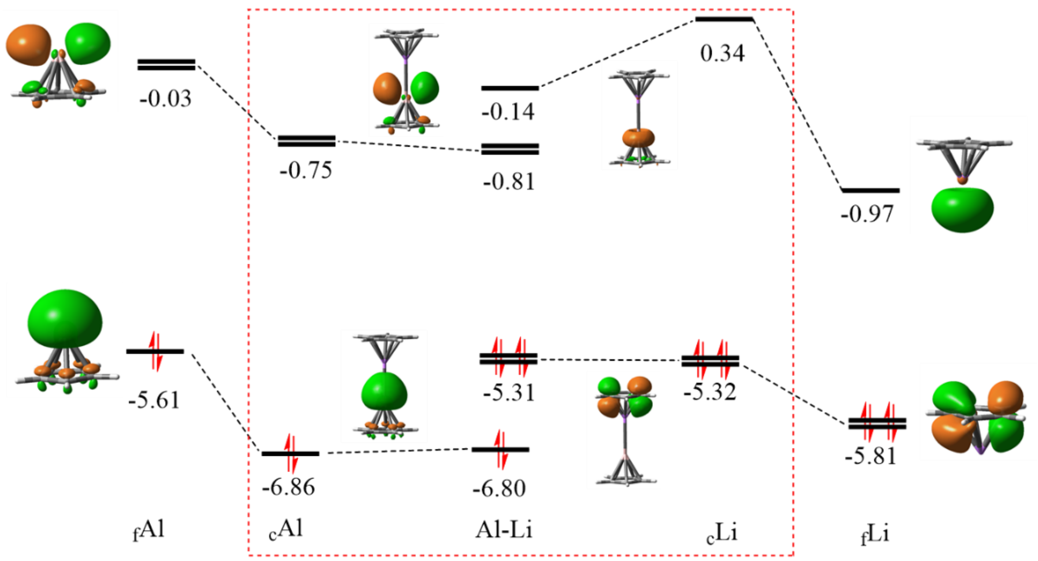

Figure S3. "*In-situ*" orbital correlation diagram with **B3LYP** functional showing the ionic bond between CpAl and CpLi with isovalues = 0.05 a.u..

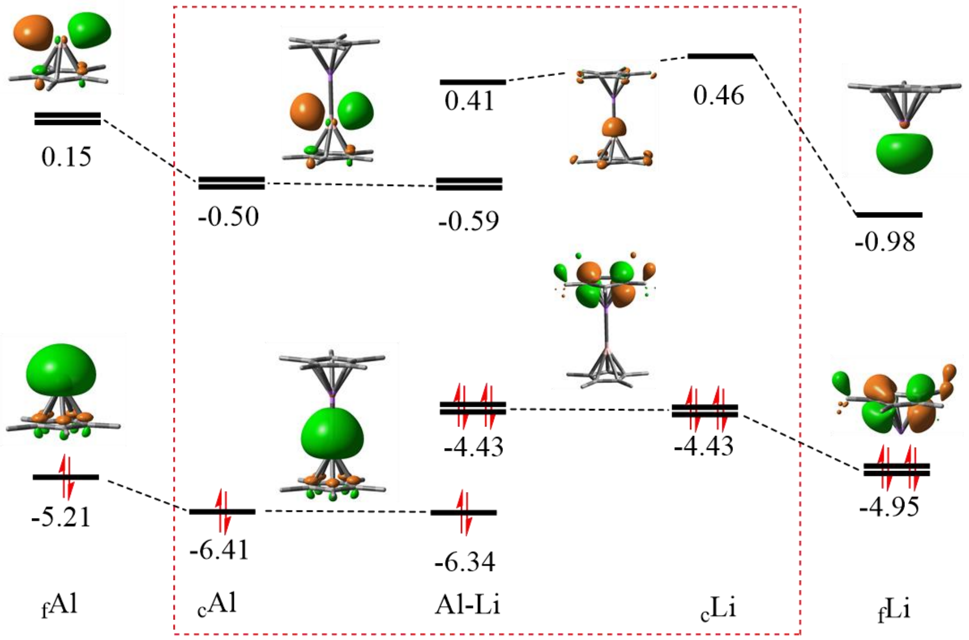

Figure S4 "*In-situ*" orbital correlation diagram with **B3LYP** functional showing the ionic bond between *CpAl and *CpLi with isovalues = 0.05 a.u..

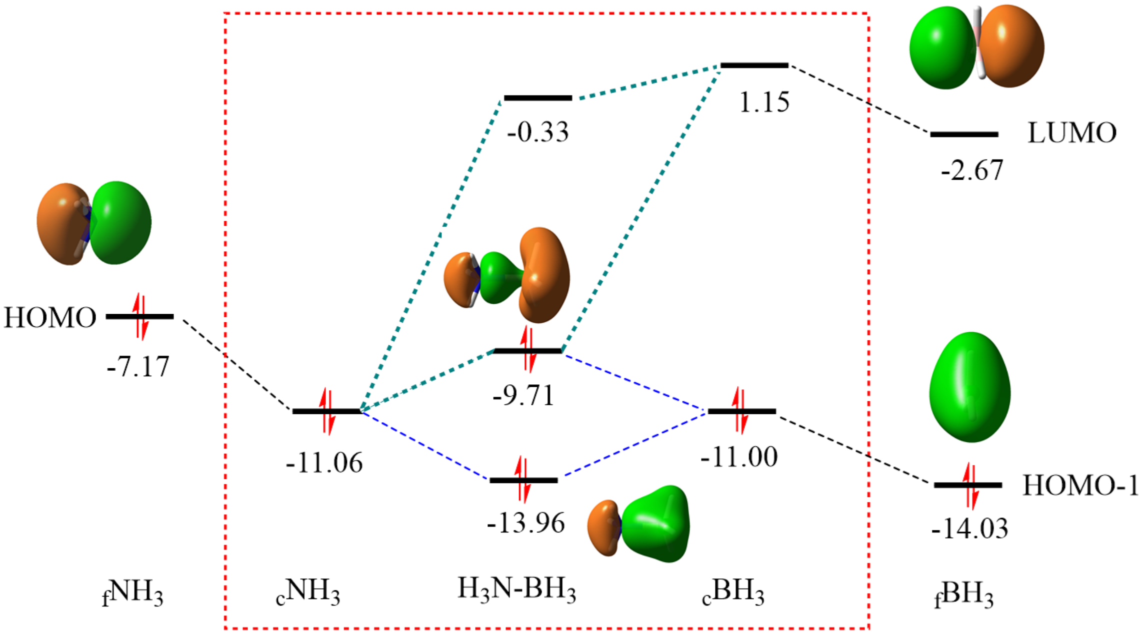

Figure S5. "*In-situ*" orbital correlation diagram with **B3LYP** functional for the dative bond in $H_3N–BH_3$ with disvalues = 0.05 a.u..

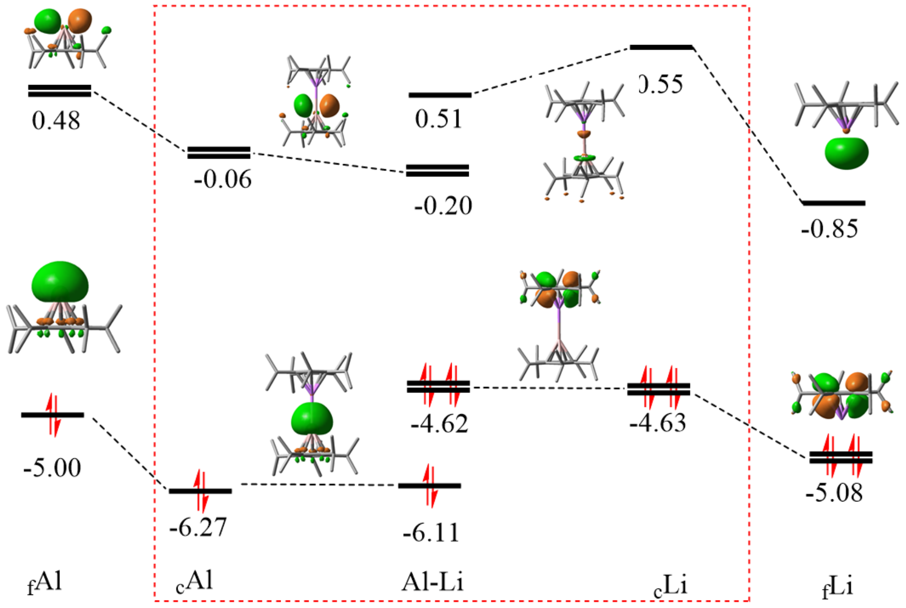

Figure S6 "*In-situ*" orbital correlation diagram with **B3LYP** functional showing the ionic bond between ^{5}CpAl and ^{5}CpLi with isovalues = 0.05 a.u..

H_3N-BH_3-DFT

N	-0.7247510000	-0.0000210000	0.0000410000
H	-1.0860080000	-0.9308740000	-0.1785170000
H	-1.0855650000	0.6200590000	-0.7170290000
H	-1.0861260000	0.3109450000	0.8953420000
B	0.9253000000	-0.0000130000	0.0000340000
H	1.2345620000	-0.3821300000	-1.1011320000
H	1.2347380000	1.1446770000	0.2195050000
H	1.2351530000	-0.7624630000	0.8813750000

H_3N-BH_3-DFT

N	-0.9964966613	0.0000149536	-0.0000845422
H	-1.3662071913	-0.9253347427	-0.1775562860
H	-1.3662266910	0.6165101562	-0.7126067278
H	-1.3659729248	0.3089081187	0.8901638854
B	1.3611648284	0.0000341409	-0.0000522760
H	1.4604208481	-0.3883703455	-1.1186679765
H	1.4603168784	1.1629994583	0.2228448552
H	1.4603039134	-0.7745817394	0.8955780680

^{5}CpAl-Li^{5}Cp-DFT

Li	-1.47374000	-0.00117900	-0.00110500
C	-3.17135700	0.13960700	1.20191900
C	-3.17185300	1.18550700	0.23725800
C	-3.17270600	0.59121400	-1.05560900
C	-3.17225400	-0.82207300	-0.88991100
C	-3.17169000	-1.10113700	0.50536100
C	-3.17453200	0.33489700	2.70403800
H	-3.17428200	1.40848200	2.88193400
C	-4.45268800	-0.19036400	3.36798100
H	-4.46537900	0.05604700	4.43196500
H	-4.55280200	-1.27146500	3.27524600
H	-5.32867500	0.26075200	2.90027200
C	-1.90896200	-0.19336500	3.39172900
H	-1.93640400	0.00260300	4.46623900
H	-1.02388900	0.30052600	2.97929000
H	-1.77961500	-1.26735300	3.25141500
C	-3.17480800	2.67459000	0.51537200
H	-3.17533900	3.17525200	-0.45089900
C	-4.45201900	3.14449700	1.22134000
H	-4.46540700	4.23284100	1.31248300
H	-4.54959900	2.72558900	2.22257600
H	-5.32906000	2.83656700	0.65075700

C	-1.90865200	3.16613700	1.22881600
H	-1.93611800	4.24879900	1.37323500
H	-1.02378600	2.92614800	0.63142400
H	-1.77857800	2.70224300	2.20747100
C	-3.17868700	1.31676900	-2.38537500
H	-3.18137100	0.55364100	-3.16127900
C	-4.45668400	2.13360300	-2.60962900
H	-4.47213800	2.55939100	-3.61531900
H	-4.55354900	2.95417100	-1.89918000
H	-5.33315500	1.49496500	-2.49329600
C	-1.91295600	2.14746400	-2.63386500
H	-1.94439500	2.62482300	-3.61615700
H	-1.02858500	1.50399200	-2.59797500
H	-1.77871500	2.93041600	-1.88626600
C	-3.17655600	-1.86268500	-1.99070000
H	-3.17654400	-2.83639300	-1.50459300
C	-4.45512000	-1.82693300	-2.83601800
H	-4.46884300	-2.65191000	-3.55167600
H	-4.55470300	-0.89795200	-3.39680600
H	-5.33096700	-1.91594500	-2.19215000
C	-1.91128400	-1.83980700	-2.85811600
H	-1.94103400	-2.62731800	-3.61485200
H	-1.02614200	-2.00134300	-2.23530100
H	-1.77972300	-0.88702200	-3.37254900
C	-3.17385300	-2.46944400	1.15506200
H	-3.17472500	-2.30786500	2.23131500
C	-4.45043500	-3.26532100	0.85932400
H	-4.46266800	-4.20035800	1.42366300
H	-4.54836800	-3.51284300	-0.19733800
H	-5.32783900	-2.68214400	1.14157800
C	-1.90658800	-3.28434500	0.86561200
H	-1.93374300	-4.24727500	1.38111100
H	-1.02292600	-2.73920400	1.21119800
H	-1.77485500	-3.47915500	-0.19953800
Al	1.15594400	-0.00124400	-0.00166700
C	1.86615900	0.17151400	-3.44821700
H	1.96798500	-0.00393000	-4.52020300
H	0.95928300	-0.33488800	-3.10946100
H	1.71752600	1.24109700	-3.29808300
C	4.41066400	0.14338000	-3.30623400
H	4.47673100	-0.13784200	-4.35813100
H	4.50275600	1.22720500	-3.24642900
H	5.26244800	-0.29124000	-2.78140000
H	1.96970300	-4.30049800	-1.39098900

H	0.96007300	-3.05903700	-0.64489100
H	1.72179200	-2.75496600	-2.20098300
C	4.41239100	-3.10016600	-1.15423300
H	4.47946400	-4.18760400	-1.20813300
H	4.50496800	-2.71187800	-2.16784300
H	5.26341800	-2.73268400	-0.57925300
C	1.86329600	-2.16793000	2.68600600
H	1.96391000	-2.65553100	3.65679800
H	0.95408500	-1.56251900	2.70687700
H	1.71968300	-2.94584700	1.93575100
C	4.40752900	-2.05356100	2.59607700
H	4.47168100	-2.44359500	3.61278200
H	4.50387800	-2.89538900	1.91136100
H	5.25797800	-1.39119000	2.42906200
C	1.85266000	1.88685700	2.88556200
H	1.95310600	2.65290200	3.65589800
H	0.94892700	2.10439700	2.31140800
H	1.69881200	0.92981800	3.38463000
C	4.39845400	1.83321600	2.76370100
H	4.46028100	2.68018700	3.44833500
H	4.49090100	0.92240400	3.35409000
H	5.25234700	1.87743400	2.08632000
C	1.85900800	3.33062400	-0.90141500
H	1.95777500	4.29649800	-1.39902600
H	0.95315900	2.84964200	-1.27776000
H	1.71082700	3.51703800	0.16249800
C	4.40389100	3.19303000	-0.88694100
H	4.46743700	4.10653100	-1.47973000
H	4.49599200	3.47158600	0.16219300
H	5.25697100	2.56146600	-1.13850600
C	3.05261100	-0.15391000	-1.20514800
C	3.05285500	-1.19300900	-0.22474600
C	3.05053600	-0.58175500	1.06651600
C	3.04911000	0.83529000	0.88402800
C	3.05062800	1.09965000	-0.51987700
C	3.09485200	-0.36452800	-2.70588700
H	3.09113300	-1.43978600	-2.86660100
C	3.09556900	-2.68546800	-0.48798800
H	3.09021900	-3.17025800	0.48517500
C	1.86811200	-3.22638800	-1.22888500
C	3.09002700	-1.29363000	2.40460000
H	3.08098100	-0.51853900	3.16705800
C	3.08513300	1.88766400	1.97487100
H	3.08081200	2.85249500	1.47361500

C	3.08947400	2.46213100	-1.18359800
H	3.08515700	2.28359500	-2.25613200

^{5}CpAl-Li^{5}Cp-BLW

LI	-1.5331166521	-0.0101149291	-0.0029694385
C	-3.2383564418	0.1318694784	1.2048144401
C	-3.2378664660	1.1874017195	0.2435690673
C	-3.2407671717	0.5993215091	-1.0570478176
C	-3.2447075450	-0.8196389887	-0.8997606755
C	-3.2435687831	-1.1085717279	0.4982291844
C	-3.2539723853	0.3170503282	2.7119504856
H	-3.2512303779	1.4000092814	2.8928402447
C	-4.5431434274	-0.2043005205	3.3621328360
H	-4.5706180342	0.0449354048	4.4350844096
H	-4.6452171318	-1.2955700047	3.2703414799
H	-5.4216031526	0.2493737691	2.8794511247
C	-1.9944592416	-0.2171552186	3.4090764905
H	-2.0334350614	-0.0339158044	4.4951379976
H	-1.0968031312	0.2856324382	3.0126369667
H	-1.8608290499	-1.2990781483	3.2574376770
C	-3.2602527388	2.6779769573	0.5334287450
H	-3.2651902959	3.1853879624	-0.4401805014
C	-4.5496167937	3.1271550397	1.2351992505
H	-4.5834505230	4.2244369252	1.3297390323
H	-4.6452110449	2.7022161351	2.2451421046
H	-5.4282708084	2.8028389242	0.6578962970
C	-2.0014145057	3.1838815208	1.2522526645
H	-2.0462556397	4.2733660240	1.4123509669
H	-1.1041736247	2.9664968912	0.6494235558
H	-1.8627480934	2.7074222990	2.2345943007
C	-3.2619051489	1.3364833371	-2.3845554405
H	-3.2617430711	0.5682378077	-3.1689898626
C	-4.5528054770	2.1393436550	-2.5983637562
H	-4.5841031170	2.5701479558	-3.6120409681
H	-4.6536978166	2.9667594242	-1.8808356667
H	-5.4299772693	1.4871630512	-2.4729540825
C	-2.0040867617	2.1799874201	-2.6372266280
H	-2.0456620130	2.6706813884	-3.6231576907
H	-1.1056663317	1.5412359065	-2.6136204678
H	-1.8698499486	2.9654643503	-1.8780971457
C	-3.2699081708	-1.8546371598	-2.0106483694
H	-3.2736702075	-2.8375788134	-1.5214189716
C	-4.5604226033	-1.8045048641	-2.8404839189
H	-4.5961233905	-2.6365472248	-3.5619645579

H	-4.6559201099	-0.8669139346	-3.4075118997
H	-5.4381986561	-1.8806056995	-2.1815446373
C	-2.0121524952	-1.8402469650	-2.8912130160
H	-2.0607502668	-2.6217674401	-3.6667595609
H	-1.1146904762	-2.0265688241	-2.2783816448
H	-1.8703696075	-0.8739314804	-3.3985070780
C	-3.2658709724	-2.4847404745	1.1399370677
H	-3.2630271472	-2.3225722995	2.2258668529
C	-4.5587146363	-3.2576760324	0.8439721973
H	-4.5906124153	-4.2018436178	1.4111018991
H	-4.6618686449	-3.5053901189	-0.2226677504
H	-5.4343236284	-2.6547218590	1.1272287308
C	-2.0104698781	-3.3191002930	0.8475142756
H	-2.0532887273	-4.2941296124	1.3594497045
H	-1.1097692862	-2.7901360502	1.2006284441
H	-1.8791908413	-3.5120943017	-0.2280426752
AL	1.2333931639	-0.0053825406	-0.0011789445
C	1.9574401859	0.1500844544	-3.4609585382
H	2.0582696374	-0.0410676784	-4.5399592094
H	1.0422795940	-0.3536198337	-3.1128420055
H	1.8086046619	1.2316570443	-3.3259835266
C	4.5068651888	0.1293173287	-3.3076344122
H	4.5830659652	-0.1635625654	-4.3656992334
H	4.5972175459	1.2239994262	-3.2584742005
H	5.3650682719	-0.3000250922	-2.7692570551
H	2.0675485677	-4.3305243661	-1.3696593569
H	1.0470468455	-3.0719066012	-0.6340769160
H	1.8206573769	-2.7811024125	-2.2031763909
C	4.5142037112	-3.1064414100	-1.1345063598
H	4.5903993838	-4.2032150214	-1.1831877215
H	4.6108165096	-2.7207029300	-2.1596024829
H	5.3690664618	-2.7277135887	-0.5544540056
C	1.9576106913	-2.1614362342	2.7113350965
H	2.0572608647	-2.6391644144	3.6976427836
H	1.0384464939	-1.5553899338	2.7211326445
H	1.8173110351	-2.9590481256	1.9668835749
C	4.5065552824	-2.0387215490	2.6107504695
H	4.5803672691	-2.4230684532	3.6393028626
H	4.6050171701	-2.8950166087	1.9280926535
H	5.3616069452	-1.3698712822	2.4312866229
C	1.9445357115	1.9091955838	2.8894035633
H	2.0407240066	2.6973853453	3.6512842336
H	1.0289398480	2.1083021523	2.3111310735
H	1.8000489588	0.9531928186	3.4141557780

C	4.4945871780	1.8517059330	2.7545659623
H	4.5652473621	2.7113677760	3.4379978359
H	4.5902867540	0.9380860447	3.3587527876
H	5.3529005806	1.8874908727	2.0670297291
C	1.9475664739	3.3395400393	-0.9212304391
H	2.0458368472	4.3070583942	-1.4362064515
H	1.0315214226	2.8525477356	-1.2898418625
H	1.8022385079	3.5446979301	0.1497083227
C	4.4971379501	3.1900217715	-0.9046952510
H	4.5694636612	4.1063452722	-1.5098528062
H	4.5917844224	3.4808695198	0.1514350021
H	5.3551084324	2.5466228813	-1.1508737554
C	3.1361153373	-0.1591816845	-1.2059362542
C	3.1381554883	-1.1968264048	-0.2187467971
C	3.1344710248	-0.5787016627	1.0731692901
C	3.1307244939	0.8409536359	0.8842813485
C	3.1313294129	1.1002520386	-0.5241131752
C	3.1869200923	-0.3757776077	-2.7092266675
H	3.1865217066	-1.4620400777	-2.8608558025
C	3.1904032341	-2.6933723334	-0.4769841474
H	3.1836811588	-3.1727822437	0.5094536081
C	1.9651096396	-3.2456347229	-1.2165486349
C	3.1828711696	-1.2864977781	2.4171299406
H	3.1735734340	-0.4964613573	3.1778330327
C	3.1746580605	1.9002228313	1.9729735306
H	3.1682959179	2.8679124730	1.4568355397
C	3.1767391750	2.4633363231	-1.1945012772
H	3.1712579511	2.2720423917	-2.2744838775

***CpAl-Li*Cp-DFT**

Li	0.00000000	0.00000000	-1.52510219
C	-0.70895031	0.97578640	-3.23882089
C	-1.14710570	-0.37271724	-3.23882089
C	0.70895031	0.97578640	-3.23882089
C	1.14710570	-0.37271724	-3.23882089
C	-0.00000000	-1.20613832	-3.23882089
C	-1.58954287	2.18781807	-3.31734570
H	-1.79485833	2.47041056	-4.35377077
H	-2.55358665	2.01873969	-2.83562078
H	-1.13083386	3.05243009	-2.83562078
C	-2.57193440	-0.83567214	-3.31734570
H	-2.90414179	-0.94361287	-4.35377077
H	-2.70903720	-1.80478035	-2.83562078
H	-3.25248041	-0.13223415	-2.83562078

C	-0.00000000	-2.70429186	-3.31734570
H	0.87930958	-3.13415529	-2.83562078
H	-0.87930958	-3.13415529	-2.83562078
H	-0.00000000	-3.05359538	-4.35377077
C	2.57193440	-0.83567214	-3.31734570
H	2.70903720	-1.80478035	-2.83562078
H	2.90414179	-0.94361287	-4.35377077
H	3.25248041	-0.13223415	-2.83562078
C	1.58954287	2.18781807	-3.31734570
H	2.55358665	2.01873969	-2.83562078
H	1.79485833	2.47041056	-4.35377077
H	1.13083386	3.05243009	-2.83562078
Al	0.00000000	0.00000000	1.20715495
C	0.71121314	-0.97890091	3.11461243
C	1.15076704	0.37390688	3.11461243
C	-0.71121314	-0.97890091	3.11461243
C	0.00000000	1.20998807	3.11461243
C	-1.15076704	0.37390688	3.11461243
C	0.00000000	2.70694447	3.15576224
H	0.87934114	3.12037849	2.66426925
H	-0.87934114	3.12037849	2.66426925
H	0.00000000	3.06348466	4.18768383
C	2.57445718	0.83649185	3.15576224
H	2.91354705	0.94666882	4.18768383
H	3.23938765	0.12794686	2.66426925
H	2.69592494	1.80055310	2.66426925
C	1.59110204	-2.18996408	3.15576224
H	1.80067110	-2.47841116	4.18768383
H	1.12271053	-3.04130298	2.66426925
H	2.54551438	-2.00757547	2.66426925
C	-1.59110204	-2.18996408	3.15576224
H	-1.12271053	-3.04130298	2.66426925
H	-1.80067110	-2.47841116	4.18768383
H	-2.54551438	-2.00757547	2.66426925
C	-2.57445718	0.83649185	3.15576224
H	-3.23938765	0.12794686	2.66426925
H	-2.91354705	0.94666882	4.18768383
H	-2.69592494	1.80055310	2.66426925

***CpAl-Li*Cp-BLW**

LI	-0.0000205314	0.0012880446	-1.5893006503
C	-0.7138061176	0.9780670246	-3.3013582180
C	-1.1507031627	-0.3765834475	-3.3004268041
C	0.7095585587	0.9811565385	-3.3013349297

C	1.1523264851	-0.3715863199	-3.3004117029
C	0.0026275470	-1.2107243389	-3.2997917865
C	-1.5991629926	2.1908450107	-3.3743128927
H	-1.8110139731	2.4807206401	-4.4177421692
H	-2.5704694759	2.0172001653	-2.8867225563
H	-1.1378200589	3.0632074213	-2.8869941903
C	-2.5778299340	-0.8436814875	-3.3726148129
H	-2.9192965016	-0.9561956794	-4.4158693486
H	-2.7130361949	-1.8207255980	-2.8843139450
H	-3.2646792481	-0.1349006107	-2.8855713151
C	0.0059113128	-2.7123629028	-3.3704030965
H	0.8938285407	-3.1420680060	-2.8822554687
H	-0.8800981842	-3.1459707818	-2.8822376579
H	0.0066961564	-3.0730959280	-4.4132511418
C	2.5814678178	-0.8324995979	-3.3726705314
H	2.7209269246	-1.8090066957	-2.8844884789
H	2.9233788718	-0.9434182706	-4.4159506132
H	3.2652829141	-0.1208239641	-2.8855839828
C	1.5896201898	2.1977759054	-3.3741325533
H	2.5616311494	2.0282930144	-2.8864769957
H	1.8002767125	2.4886974409	-4.4175120187
H	1.1244141101	3.0680400409	-2.8867372831
AL	-0.0000176142	0.0010531183	1.2546493739
C	0.7116465164	-0.9840598390	3.1685373078
C	1.1557352096	0.3726494569	3.1693553098
C	-0.7159259897	-0.9809494913	3.1685600737
C	0.0026481241	1.2142662072	3.1698209508
C	-1.1540996798	0.3776775118	3.1693745316
C	0.0059252109	2.7143803427	3.2135559801
H	0.8939241648	3.1303766538	2.7183647793
H	-0.8802405143	3.1342705874	2.7183667523
H	0.0067139405	3.0733036993	4.2548619278
C	2.5834938052	0.8330741482	3.2129761783
H	2.9252375468	0.9426989542	4.2542904504
H	3.2535487587	0.1174040408	2.7173466817
H	2.7090596030	1.8058635914	2.7182862991
C	1.5907907810	-2.1996322595	3.2107807830
H	1.8011525063	-2.4916970897	4.2517376148
H	1.1169957421	-3.0575962177	2.7145720716
H	2.5545081131	-2.0178154640	2.7158054835
C	-1.6003732548	-2.1926754706	3.2109087335
H	-1.1303727015	-3.0527639552	2.7147702666
H	-1.8119764924	-2.4837204374	4.2518998389
H	-2.5633089388	-2.0067030743	2.7159576003

C	-2.5798350862	0.8443150486	3.2129693874
H	-3.2529936422	0.1314897085	2.7174438305
H	-2.9210839760	0.9555712057	4.2542729493
H	-2.7011630485	1.8175724063	2.7181429878

CpAl-LiCp DFT

Li	-0.00331030	-1.62769972	-0.00000000
C	0.37180978	-3.34859386	1.14260570
C	1.20198069	-3.34672635	-0.00000000
C	-0.97137830	-3.35149573	0.70620425
C	-0.97137830	-3.35149573	-0.70620425
C	0.37180978	-3.34859386	-1.14260570
H	0.70511824	-3.36358953	2.16859801
H	0.70511824	-3.36358953	-2.16859801
H	2.28078982	-3.35967390	-0.00000000
H	-1.84409028	-3.36877993	1.34027213
H	-1.84409028	-3.36877993	-1.34027213
Al	-0.00262075	1.12765625	0.00000000
C	-0.97232452	3.07610015	-0.70699884
C	-0.97232452	3.07610015	0.70699884
C	0.37233682	3.07378991	-1.14384341
C	0.37233682	3.07378991	1.14384341
C	1.20342053	3.07231374	0.00000000
H	2.28089465	3.05538677	0.00000000
H	0.70527627	3.05812067	-2.16859992
H	-1.84401248	3.06261136	-1.34038824
H	-1.84401248	3.06261136	1.34038824
H	0.70527627	3.05812067	2.16859992

CpAl-LiCp BLW

LI	1.6758978231	0.0617192672	0.0623350592
C	3.3342762489	-1.0180180614	-0.6464961287
C	3.3916303053	-0.9294969845	0.7618528785
C	3.3637056977	0.2941409170	-1.1678557569
C	3.4391520892	1.1938767692	-0.0817579403
C	3.4565432763	0.4374962731	1.1109666288
H	3.5258605610	0.8337575666	2.1122330720
H	3.4022137409	-1.7607991915	1.4497368592
H	3.2928977103	-1.9288987940	-1.2235426231
H	3.3487870714	0.5617528899	-2.2131175224
H	3.4928760232	2.2693321788	-0.1515467321
AL	-1.1741255261	0.0561770705	0.0593480584
C	-3.1584925992	1.1921922205	-0.0776550649
C	-3.0933649798	0.2966828912	-1.1696580943

C	-3.1733124416	0.4293181134	1.1125902598
C	-3.0679107836	-1.0194953915	-0.6544607806
C	-3.1173595205	-0.9376055441	0.7559995698
H	-3.0980930130	-1.7703008582	1.4399177070
H	-3.2045506965	0.8214584310	2.1159749156
H	-3.1762576402	2.2679349501	-0.1407580820
H	-3.0521385350	0.5700251068	-2.2113316437
H	-3.0036648117	-1.9255908199	-1.2344296393

[1] D. Cooper, *Valence bond theory* (Elsevier, 2002).
[2] S. S. Shaik and P. C. Hiberty, *A chemist's guide to valence bond theory* (John Wiley & Sons, 2007).
[3] W. Wu, P. Su, S. Shaik, and P. C. Hiberty, Chem. Rev. **111**, 7557 (2011).
[4] Y. Mo and S. D. Peyerimhoff, J. Chem. Phys. **109**, 1687 (1998).
[5] Y. Mo, L. Song, and Y. Lin, J. Phys. Chem. A **111**, 8291 (2007).
[6] W. Chen and M. S. Gordon, The Journal of Physical Chemistry **100**, 14316 (1996).
[7] B. Jeziorski, R. Moszynski, and K. Szalewicz, Chemical Reviews **94**, 1887 (1994).
[8] M. O. Sinnokrot and C. D. Sherrill, Journal of the American Chemical Society **126**, 7690 (2004).
[9] Y. Geng, T. Takatani, E. G. Hohenstein, and C. D. Sherrill, The Journal of Physical Chemistry A **114**, 3576 (2010).
[10] C. D. Sherrill, Accounts of Chemical Research **46**, 1020 (2013).
[11] M. P. Mitoraj, A. Michalak, and T. Ziegler, Journal of Chemical Theory and Computation **5**, 962 (2009).
[12] T. Ziegler, A. Rauk, and E. J. Baerends, Theoretica chimica acta **43**, 261 (1977).
[13] P. Su, Z. Jiang, Z. Chen, and W. Wu, The Journal of Physical Chemistry A **118**, 2531 (2014).
[14] P. Su, Z. Tang, and W. Wu, WIREs Computational Molecular Science **10**, e1460 (2020).
[15] Z. Tang, Y. Song, S. Zhang, W. Wang, Y. Xu, D. Wu, W. Wu, and P. Su, Journal of Computational Chemistry **42**, 2341 (2021).
